# Estimated annual increments in stature and weight among soccer players 11–18 years

**DOI:** 10.5114/biolsport.2025.144294

**Published:** 2024-10-23

**Authors:** Robert M. Malina, Diogo V. Martinho, Tomas Oliveira, Jorge M. Celis-Moreno, António J. Figueiredo, Andre Seabra, Jan M Konarski, Sean P. Cumming, Manuel J. Coelho-e-Silva

**Affiliations:** 1 University of Texas at Austin, Department of Kinesiology and Health Education, Austin, Texas, United States of America; 2 University of Louisville, School of Public Health and Information Sciences, Louisville, Kentucky, United States of America; 3 University of Coimbra, CIDAF (uid/04213), Coimbra, Portugal; 4 University of Coimbra, FCDEF, Coimbra, Portugal; 5 Interactive Technologies Institute, Laboratory of Robotics and Engineering Systems, Funchal, Portugal; 6 University of Santo Tomas, Bogotá, Colombia; 7 Portugal Football School, Portuguese Football Federation, Lisbon Portugal; 8 University of Porto, Faculty of Sport, CIAFEL, Porto, Portugal; 9 Poznań University of Physical Education, Sports Science, Poznań, Poland; 10 University of Bath, Department for Health, Bath, UK

**Keywords:** Adolescent spurt, Youth athletes, Growth status, Skeletal maturation, Individual differences

## Abstract

Annual increments in height and weight were estimated in a mixed-longitudinal sample of Portuguese male soccer players 11–14 years at baseline. This study aims to compare estimated increments with reference values for the general population and also with estimates from youth soccer literature. The sample included 87 under-13 (U13) and 72 under-15 (U15) players. Allowing for drop-outs and/or transfers, participants were followed over five seasons. Annual measurements of height and weight were taken for the total mixedlongitudinal sample of 571 players, from which annual increments were calculated for 409 occasions. Mean heights and weights of the sample and median annual increments were compared with corresponding estimates for the general population in addition to soccer players based on studies spanning 2000–2015. Mean heights of the sample were slightly but consistently less than the reference, while mean weights were below the reference at 11–13 years and approximated the reference at 14–18 years. Median annual height and weight increments of the soccer players approximated the respective medians for the general population, but increments among individual players varied considerably with age. In contrast, the heights and weights of the Portuguese players were below the means for the composite sample of soccer players except at 18 years, but estimated increments were similar to the composite sample. In summary, increments in the heights and weights of the Portuguese players approximated the general population and means for other soccer players, while mean heights were consistently less than the reference except at 18 years.

## INTRODUCTION

The growth status of youth soccer players is reasonably well documented [1]. Mean heights and weights of players 9–18 years in studies spanning 2000 to 2015 overlapped considerably, and were, on average, greater compared to studies spanning 1978 through 1999. Corresponding data on the timing of the adolescent growth spurt are limited. Mean ages at peak height velocity (PHV) in two longitudinal samples of school and local level European soccer players in the 1980s were 14.1 and 14.2 years, while ages at PHV among players at several professional clubs in Europe between 2000 and 2020, with one exception, ranged from 13.4 to 14.2 years [2]. Allowing for variation in methods of estimating age at PHV, the limited observations suggest negligible secular change in this indicator of maturity timing among soccer players.

In addition to variation in timing of the adolescent spurt among individuals [2], adolescence is associated with rapid development of specific functional capacities some of which have a well-defined spurt [3], and with heightened risk for overuse and growth-related injuries [4]. Individual differences in responses to training stimuli during adolescence are also considerable [5]. In this context, coaches, trainers and medical practitioners working with youth athletes in general [6] and specifically with soccer players [7] are interested in monitoring growth rates of individual players in an effort to prescribe developmentally appropriate training loads and content, to avoid or perhaps better assess risk for injury, and to evaluate athletic aptitude and potential relative to the adolescent growth spurt, specifically the initiation of the spurt (take-off) and the interval of maximum rate of growth in height (PHV).

Monitoring height and weight is increasingly common in many youth sport programs. Measurements of height and weight, and in turn estimated increments, are influenced by inter- and intra-individual variability in the measurement process and also by diurnal variation. Measurements of height taken after a period of intensive physical activity or training are commonly less due to compression of inter-vertebral cartilage than those taken after a period of rest [2]. An additional concern is seasonal variation. Studies in Europe and North America indicate that children and adolescents grow more in height during the spring and summer, and more in weight in the fall and winter [8]. The preceding factors have implications for estimates of increments in height and weight, especially based on measurements over relatively short intervals. They also have implications for decisions to adjust player evaluations, training or competition relative to growth and/or maturity status (i.e., maturity-matching or biobanding). Estimates of growth rate are also affected by the reliability of the measurements [9].

Given the increased interest in monitoring the estimated rates of growth in individual athletes [6, 7], the purpose of this study is twofold. It initially considers estimated annual increments (rates of growth) in height and weight based on annual observations spanning the interval of adolescence in a mixed-longitudinal sample of male soccer players, and then compares the estimated increments with reference values for the general population and also with estimated increments in height and weight derived from a recent review of the growth status of youth soccer players.

## MATERIALS AND METHODS

### Study Design

The data are part of the *Coimbra Soccer Longitudinal Project* [10], which followed standards established by the declaration of Helsinki [11]. Formal approval was obtained from the *University of Coimbra Sports Sciences and Physical Education Board* (CE/FCDEFUC/00122014) and included agreements with the respective clubs involved. Written consent was obtained from parents/legal guardians of each player; players were also informed that participation was voluntary and that they could withdraw from the study at any time.

### Participants

The study was mixed-longitudinal. The baseline sample included 87 under-13 (U13) and 72 under-15 (U15) players from five clubs in and around the city of Coimbra in the midlands of Portugal. The clubs competed in a 9-month season (September through May with a two week break over the Christmas/New Year holiday) through the Portuguese Soccer Federation. All players except one were of European ancestry. Experience in soccer spanned 1 to 6 (median 3) years among U13 and from 2 to 8 (median 5) years among U15 players. Players trained three times per week (about 90 minutes per session) and participated in one game per week during the season, usually on Saturday. The sample, specifically the players persisting at their respective clubs, were subsequently observed annually within a two week period in December (U13 players) and in April (U15 players) over five consecutive years.

### Anthropometry

Measurements of height and weight were taken annually using the same protocol. Players wore shorts and a t-shirt; shoes were removed. Stature was measured to 0.1 cm (Harpenden 98.603 stadiometer, Holtain Ltd), and weight to 0.1 kg (SECA 770 scale). Heights and weights were measured by a single trained observer. Intra-observer technical errors of measurement were 0.27 cm for height and 0.47 kg for weight [12], and were within the range of technical errors noted in studies of the general population and youth athletes [13].

### Skeletal age (SA)

Chronological ages (CA) were calculated as the difference between date of birth and date at each observation. CAs at baseline ranged from 10.98 to 12.94 years among U13 and from 13.30 to 15.25 years among U15 players (note, all U15 players were < 15.0 years at the start of the season). Radiographs of the left hand-wrist were taken at observations 1, 3 and 5 for U13 players and at observations 1 and 3 for U15 players for the purposes of assessing skeletal maturity status. Skeletal age (SA) was assessed with the Fels method [14]. An SA was not assigned to skeletally mature players; they were simply noted as such as the CA at which maturity was attained was not known.

### Statistical analysis

*Longitudinal Sample.* Annual measurements were available for 57 players on five occasions, 38 on four occasions, 20 on three occasions, and 27 on two occasions, yielding a total of 409 annual increments spanning 11–18 years. The subsample of 20 players with annual observations on three occasions included three players who were measured in the 1^st^, 2^nd^, 3^rd^ and 5^th^ seasons. Means and standard deviations for CA, height and weight of the total mixedlongitudinal sample were calculated by single year CA groups defined by the whole year (11 refers to 11.00 to 11.99 years, 12 refers to 12.00–12.99 years, etc.), and also for the subsample of players with SA assessments. Means, standard deviations and medians were calculated for estimated increments between annual measurements of individual players observed on two or more occasions. Estimated increments between annual measures were adjusted for the CA interval between observations. Analyses were done with SPSS version 20.0 (SPSS Inc, IBM Company, N.Y., USA). The longitudinal height records for 58 of the 87 U13 players with four or five observations were modeled with the Superimposition by Translation and Rotation (SITAR) method [15, 16, 17] to estimate age a PHV. The respective estimate was 13.6 ± 0.9 years [18].

*European Reference Data.* Reference data for growth status and estimated rates of growth are lacking for Portuguese youth. Median heights and weights of a recent sample of Swiss boys [19] were thus used as a reference for size attained, while median annual increments in heights and weights of Swiss boys in the first Zurich Longitudinal Study, 1954– 1976 [20] were used as the reference for rate of growth. Mean heights and weights of the mixed-longitudinal sample of soccer players were thus plotted relative to the reference medians for Swiss boys, while median annual increments in height and weight were plotted relative to the respective medians for the Zurich Longitudinal Study.

*U.S. Reference Data for Estimated Velocities.* As the Zurich reference for growth velocities is somewhat dated, the estimated annual increments of the soccer players were also plotted relative to median increments in height for a recent sample of U.S. children and adolescents [21]. The U.S. reference was based on participants in a longitudinal study of bone mineral in density among children and adolescents followed longitudinally for up to seven years at five clinical centers; reference values for increments in body weight, however, were not reported.

*Comparative Data for Youth Soccer Players.* Heights and weights, and estimated velocities of growth in height and weight for a composite sample of soccer players based on 121 studies spanning 2000–2015 were estimated [1]. The 121 studies reported 218 means for height, weight and CA of players from 33 countries: Europe (148 means, 67.9%); Americas (41, 18.8%); Asia (11, 5.0%); Middle East (10, 4.6%); Africa (7, 3.2%); New Zealand (1 mean, 0.5%). The age-specific composite means for CA, height and weight for single year CA groups 9 through 18 years in the 121 studies were calculated and fitted with Preece-Baines model 1 [22, 23]. The model provided estimates of mean heights and weights and of mean velocities of growth in height and weight based on the composite sample of soccer players at each age from 9 through 18 years [1].

## RESULTS

**FIG. 1:**
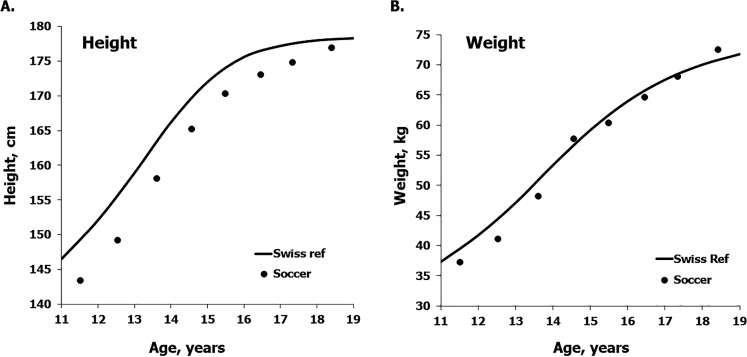
Mean heights (a) and weights (b) of the mixed-longitudinal sample of soccer players plotted relative to respective the reference medians for heights and weights of Swiss youth [20].

Descriptive statistics for the heights and weights of the mixed-longitudinal sample of soccer players 11 through 18 years are summarized in Table 1. Mean heights of the players are, on average, consistently less than reference medians for Swiss boys except at 18 years (Figure 1 – left), while mean weights are slightly below the reference at 11–13 years and approximate the reference from 14 through 18 years (Figure 1 – right).

**TABLE 1:** Means (M) and standard deviations (SD) for chronological age (CA), height and weight by single year CA groups in the total mixed-longitudinal sample of soccer players.

Age group	N	CA, yrs		Height, cm		Weight, kg	

		M	SD	M	SD	M	SD
11	62	11.5	0.3	143.4	6.0	37.3	6.0
12	79	12.5	0.3	149.2	7.1	41.1	7.1
13	101	13.6	0.3	158.1	8.6	48.2	9.2
14	116	14.6	0.3	165.2	8.1	54.7	9.1
15	101	15.5	0.3	170.3	6.3	60.4	8.8
16	60	16.4	0.3	173.1	5.7	64.6	9.2
17	33	17.3	0.3	174.8	6.1	68.1	9.8
18	19	18.4	0.5	176.9	5.2	72.5	9.5

CA (chronological age); M (mean); SD (standard deviation).

Means and standard deviations by CA group for CA and SA of non-skeletally mature players and for CA of skeletally mature soccer players in the mixed-longitudinal sample for whom radiographs were available are summarized in Table 2. SAs of the mixed-longitudinal sample are, on average, slightly in advance of CAs among players 11 through 15 years; at 16 years, mean SAs and CAs are equivalent although 11 players 16 years of age (mean CA = 16.8 years) were skeletally mature and an SA is not assigned.

**TABLE 2:** Means (M) and standard deviations (SD) by chronological age (CA) group for CAs and Fels skeletal ages (SA) of non-skeletally mature and for CAs of skeletally mature soccer players in the mixed-longitudinal sample for whom radiographs were available*.

Age group	N	Not Skeletally Mature				Skeletally Mature Age		

		CA, yrs		SA, yrs		N	CA, yrs	
	
		M	SD	M	SD		M	SD
11	62	11.5	0.3	11.8	1.4	-		
12	25	12.5	0.3	12.3	1.6	-		
13	80	13.6	0.3	14.2	1.0	-		
14	46	14.5	0.3	14.8	1.3	-		
15	52	15.5	0.3	16.2	1.2	4	15.8	0.2
16	31	16.5	0.4	16.5	1.1	11	16.8	0.3

* Radiographs were taken at observations 1, 3 and 5 for U13 players and at observations 1 and 3 for U15 players. An SA is not assigned to skeletally mature players; CA (chronological age); SA (skeletal age); M (mean); SD (standard deviation).

Descriptive statistics for estimated annual increments in height and weight are summarized in Table 3. Variation in estimated annual height and weight increments within each CA group is considerable and varies with age. The range of variation in height increments increases from 11 through 15 years and then declines, while the corresponding range of variation in weight increments is variable, and approximates 19 to 20 kg/yr among players 15 and 16 years. Of relevance to the broad range among players 15 and 16 years, is influenced by players who lost a significant amount of weight; the actual estimates range, respectively, from -5.4 to 13.9 kg/yr and from -5.7 to 14.0 kg/yr.

**TABLE 3:** Means (M), standard deviation (SD), medians (Md) and ranges (minimum, maximum) for estimated increments in height and weight between annual observations (rates of growth) by single year chronological age (CA) groups in the mixed-longitudinal sample of soccer players.

CA Midpoint^†,^yrs			Estimated Increments*									

			Height, cm/yr					Weight, kg/yr				

N	M	SD	M	SD	Md	Min	Max	M	SD	Md	Min	Max
54	12.0	0.3	6.4	2.1	6.2	3.5	11.8	4.5	2.1	3.9	1.5	10.3
65	13.1	0.3	7.4	2.2	7.2	3.9	12.7	5.9	2.7	6.1	-0.2	13.5
89	14.1	0.3	6.9	2.6	7.0	1.5	14.5	6.0	2.5	6.1	-0.5	10.3
90	15.0	0.3	4.7	2.9	4.3	0.3	15.6	5.3	3.1	5.3	-5.4	13.9
61	16.0	0.3	2.8	2.1	2.3	0.0	7.4	4.6	3.2	4.2	-5.7	14.0
32	16.9	0.3	1.1	1.7	0.7	0.0	9.8	2.9	2.2	3.0	-1.7	8.0
18	18.0	0.5	1.1	0.9	0.8	0.2	3.0	2.8	3.3	2.1	-2.1	13.4

* Estimated increments were adjusted for the observed CA interval between measurements for individual players;

† Age midpoint is the midpoint between CAs at the initial and subsequent measurement; CA (chronological age); M (mean); SD (standard deviation); Md (median); Min (minimum); Max (maximum); yr (years).

**FIG. 2:**
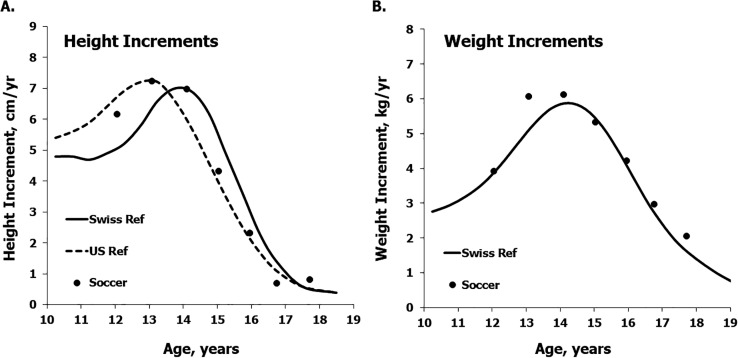
Median increments in heights (a) and weights (b) of the mixed-longitudinal sample of soccer players plotted relative to the reference medians for increments in height among Swiss [19] and U.S. [21] boys and the reference medians for increments in weight among Swiss boys [19].

Median height increments of the soccer players increase from 12 to 13–14 years and then decline through 17–18 years (Figure 2, left). The median increments approximate the U.S. reference across the age range, and are in advance of the earlier Swiss reference at 12 and 13 years. The differences between the U.S. and Swiss references at 10–16 years suggest secular variation. Corresponding median increments for body weight of the soccer players approximate the Swiss reference throughout the age range, though estimated median increments at 13 and 14 years are slightly above the reference (Figure 2, right).

**FIG. 3:**
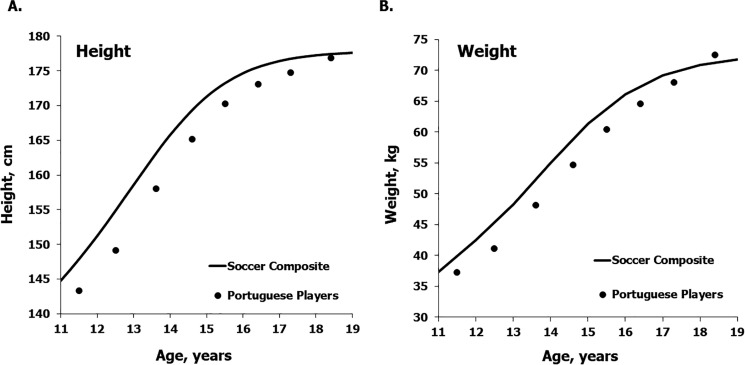
Mean heights (a) and weights (b) of the mixed-longitudinal sample of soccer players plotted relative to the respective means for a composite sample of soccer players based on the literature [1].

**FIG. 4:**
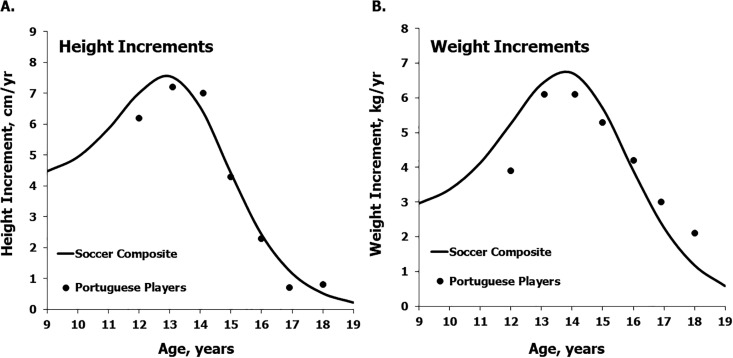
Median increments in heights (a) and weights (b) of the mixed-longitudinal sample of soccer players plotted relative to the estimated mean increments in heights and weights for the composite sample of soccer players based on the literature [1].

Means for height and weight and medians for estimated annual increments in the mixed-longitudinal sample of Portuguese soccer players are plotted relative to the corresponding estimated means for a composite sample of soccer players from the literature based on Preece-Baines model 1 in Figures 3 and 4, respectively. Mean heights and weights of the Portuguese players are consistently below the composite means except at 18 years (Figure 3 left and 3 right, respectively), while the median annual increments in heights and weights of the Portuguese players approximate the respective estimated means for the soccer players based on the literature across the age range except at 12 years (Figure 4 left and 4 right, respectively).

As noted in the methods section, the longitudinal height records of 58 U13 players in the present sample who had four or five annual observations over the five year interval of the study were modeled to estimate age at PHV for each player [18]. Using the mean age at PHV for the sample based on the SITAR model, 13.6 ± 0.9 years, the players were classified as late, average or early maturing relative to mean and standard deviation for the total sample. Based on the annual measurements of the heights of players in the subsample for which age at PHV was estimated, medians and ranges for annual increments of growth in height were calculated for the respective maturity groups and are summarized in Table 4. Estimated annual increments in height vary considerably by CA within each maturity group, but the ranges of variation in estimated increments were largest at the CA closest to the respective mean age at PHV for each of the three maturity groups.

**TABLE 4:** Medians (Md) and ranges of annual increments in height (cm/year) by chronological age (CA) groups among U13 soccer players followed over 4–5 years classified as late, average or early maturing based on age at PHV; means and standard deviations for ages at PHV in each maturity group are also indicated*.

CA interval, yrs^†^	Annual Increments in Height (cm/year) by Maturity Status								

	Late (n=11, 14.92 ± 0.35 yr)			Average (n=39, 13.54 ± 0.53 yr)			Early (n=8, 12.24 ± 0.20 yr)		

	n	Md	Range	n	Md	Range	n	Md	Range
11.0 to 11.9	6	4.9	4.2–5.5	28	6.2	4.0–10.0	6	10.0	6.3–11.8
12.0 to 12.9	11	4.7	3.9–5.3	39	8.3	5.5–11.7	8	8.7	4.9–12.7
13.0 to 13.9	10	6.6	4.3–8.6	38	8.0	3.0–13.2	8	4.3	1.5–5.4
14.0 to 14.9	5	7.6	7.1–15.6	29	4.1	0.8–8.8	7	1.0	0.3–2.3
15.0 to 15.9	4	4.8	4.7–7.4	6	1.5	0.0–3.5	2	-	0.0–1.7

* Ages at PHV for the 58 players were estimated with the SITAR model; mean age at PHV for the total sample was 13.6 ± 0.9 years [18]. A band of plus/minus 1.0 year of mean age at PHV defined average maturity status; an age at PHV more than +1.0 year of the mean indicated late maturity status, and an age at PHV more than –1.0 year of the mean indicated early maturity status;

† 12 = 12.0 to 12.9; the midpoint of the two observations is about 12.0 years, etc; CA (chronological age); Md (median).

## DISCUSSION

Mean heights of the mixed-longitudinal sample of soccer players 11–17 years from the Coimbra region of the Portugal midlands were slightly less than the reference for Swiss youth and also less than estimates for a composite sample of soccer players through adolescence, but approximated the respective references at 18 years (Figures 1 left and 3 left, respectively). Mean weights of the players were below the Swiss reference at 11–13 years and approximated the reference at 14 through 18 years (Figure 1 right), but were consistently below means for the composite sample of soccer players except at 18 years (Figure 3 right). Overall, the Portuguese and composite samples of soccer players tended to have, on average, more weight-for-height compared to the Swiss reference across the age range considered.

Mean heights of the mixed-longitudinal sample of soccer players from the Coimbra region of Portugal were also consistently less than corresponding means for soccer players 11–15 years from the Lisbon, Porto and Braga regions, while heights of the samples were similar at 16–17 years [24, 25]. The mean heights of the Coimbra players were also slightly less than reference medians [26] for Portuguese boys at 11 and 12 years, but approximated the reference medians from 13 through 16 years. The heights of the Coimbra players were also less than proposed reference medians for southern European boys 11 through 15 years, but approximated the reference at 16–17 years and were greater than the reference at 18 years [27]. The reference medians for Southern European boys were based on surveys in Spain, Italy and Greece, and were consistently shorter than reference medians for Northern European boys based on surveys in Norway, Sweden, the Netherlands, Belgium, Germany, Estonia, Lithuania and the Czech Republic [27]. Nevertheless, the trend in the heights of Coimbra and other Portuguese soccer players likely reflected the differential and selective persistence of taller players into later adolescence [1, 23, 28].

Of relevance, estimated age at PHV for the sample of professional club soccer players in the Coimbra study, 13.6 ± 0.9 years, was reasonably similar to estimated age at PHV of club soccer players in Bilbao, Spain, 13.5 ± 0.9 years. The estimates for soccer players in Portugal and Spain were, on average, slightly earlier than estimates for soccer players in several northern European countries – Denmark, Belgium, the Netherlands, England and Wales, which ranged from 13.8 ± 0.7 through 14.2 ± 0.9 years [18].

In contrast to body size per se (Figures 1 and 3), median annual increments in the heights and weights of the Coimbra soccer players generally approximated the respective references for the general population (Figure 2) and the estimates for the composite sample of soccer players (Figure 4). The variation in estimated increments among players within CA groups, however, merits attention. Ranges of estimated annual increments in each CA group (Table 3) spanned the range of the 3^rd^ and 97^th^ percentiles of the reference samples [19]. Among the players 12 and 13 years, the range of estimated increments in height was about 8 and 9 cm/year, but among players 14 and 15 years the range was about 13 and 15 cm/year. In contrast, the range of variation in estimated increments in body weight showed no consistent pattern across the age range (Table 3): relatively low at 12, 14 and 17 years (about 9–11 kg/year), larger at 13 and 18 years (about 13–15 kg/year), and largest at 15 and 16 years (about 19–20 kg/year). Several players showed negligible weight losses ranging from -0.1 to -2.1 kg, while two players experienced seemingly large weight losses followed by rapid gains. One of the latter lost 5.4 kg between 14 and 15 years, but gained 13.6 kg between 15 and 16 years, while the other lost 5.7 kg between 15 and 16 years and gained 8.3 kg between 16 and 17 years.

In the context of the preceding, it important to recognize that the range of changes in height and weight during adolescence largely reflects normal variation in the tempo of growth and maturation. However, changes in body mass may be influenced by other factors, for example, with the effects of weight training. Strength and conditioning programs are common in professional soccer academies and likely influence gains in body mass and/or changes in body composition, especially during the latter stages of puberty when both neural and structural adaptations are more likely [29]. Dietary conditions associated with a focus on excess body mass may be an additional factor.

Inter- and intra-individual variation in estimated rates of growth in height (cm/year) and weight (kg/year) merits attention as coaches and trainers are increasingly encouraged to monitor the heights and weights of youth players *over relatively short intervals* with the objective of identifying rates of growth that might be indicative of the onset and perhaps the peak of the growth spurt for the purpose of individualizing training programs [6, 30, 31]. Moreover, the Growth and Maturation Screening Programme implemented by the English Premier League recommends measurement of the heights and weights of registered academy players 9 years and older *at three-to-four month intervals* [7]. The growth status and estimated rates of individual players, in parallel with fitness measurements and injury audits, are monitored in an effort to better understand the impact of the transition into the adolescent growth spurt on fitness and performance and on the incidence and burden of injury. The data are also used in applications of bio-banding, i.e., grouping players by maturity status for training and competition [7, 32], and in efforts to identify developmentally sensitive indicators of fitness and match performance.

Estimated increments in height > 0.6 cm/month, based on monthly measurements of soccer players 11–19 years during the course of a season (September through April) were associated with an increased risk of injury [33, 34, 35]. Extrapolating the monthly increments in height through a year, an estimated velocity of growth ≥ 7.2 cm/year was suggested as indicating that a player was in his growth spurt [33, 34, 35].

It is important to note, however, that variation in rates of growth at PHV among individuals is considerable and that some players may or may not exceed this threshold. In the longitudinal subsample of 58 players with 4 or 5 observations for whom ages at PHV were estimated, annual increments in height varied considerably by CA within each maturity group (Table 4). Ranges of variation in estimated increments were largest at the CA closest to the respective mean age at PHV for each of the maturity groups defined relative to mean age at PHV in the sample. Of specific relevance, early maturing 11–12 year old players may be post-PHV and may also have an estimated rate of growth < 7.2 cm/year.

The interval of the growth spurt, of course, begins with the onset (take-off) of the spurt and continues to the cessation of growth in height in later adolescence, often considered as a height gain < 1.0 cm/year. Individual differences in estimated timing and rates of growth in height at the initiation of the growth spurt and at PHV thus merit attention. As noted in the methods, the SITAR model used to evaluate the longitudinal height records of 58 U13 soccer players also provided estimates of the CAs and rates of growth at take-off and at PHV. Estimated ages at take-off among the soccer players spanned 9.9 to 13.0 years and rates of growth at take-off spanned 3.4 to 6.1 cm/year, while estimated ages at PHV spanned 11.9 to 15.6 years and rates of growth at PHV spanned 6.7 to 13.6 cm/year [18]. Of relevance, the upper range of estimated ages at take-off and the lower range of estimated ages at PHV overlapped, while the ranges for estimated rates of growth in height at take-off and at PHV did not overlap. The upper range of estimated heights at take-off (130.0 to 153.8 cm) and the lower range of estimated heights at PHV (145.9 to 169.7 cm) also overlapped.

Variation in the timing of the growth spurt and in estimated rates of growth during the interval of the spurt among individuals is thus considerable. The variation in estimated growth rates is also especially relevant given the increasing emphasis on estimated increments in height over relatively short intervals, i.e., 3 to 4 months, and the increasing dependence upon predicted estimates of maturity timing, labeled predicted maturity offset or the time before peak height velocity, and of maturity status – percentage of predicted adult height attained at the time of observation in studies of youth athletes [9]. The following comment of Preece [36, p. 4] thus merits attention: the major source of error in the prediction of height “…is the inability to predict the timing or the intensity of the adolescent growth spurt.” The preceding must also be considered in the context of inter- and intra-individual variability in measurements of height and weight, which influence estimated increments. Distributions of increments calculated over short intervals also tend to be skewed, while short- and long-term growth velocities are, in general, poorly correlated [37, 38]. Diurnal variation in measurements of height and seasonal variation in rates of growth are also potential confounding factors. Given the latter concerns, it has been recommended that increments of growth in height should be estimated annually to eliminate the seasonal effect [8].

The present study is not without limitations. The study is limited to players in several clubs in Central Portugal, specifically the region around Coimbra. The estimates of rate of growth in height and weight were based on a mixed-longitudinal sample of U13 and U15 soccer players observed at two, three, four and five occasions. Unfortunately, the lower CA limit of the sample was 11 years, and thus did not include the transition into adolescence for some players, i.e., 9–10 years, The estimated increments or growth rates may also be influenced by differential drop-out and/or persistence of players in the respective clubs; a related factor may be the transfer of some players to other clubs outside of the region. Comparisons of estimated increments by maturity status were limited to a sample of U13 players who were observed on four or five occasions and for whom age at PHV could be estimated. Unfortunately, corresponding estimates of skeletal maturity status were limited to the first, third and fifth observations, so that comparisons of estimated rates of growth by skeletal maturity status and age at PHV across the CA range of the study were not possible.

Allowing for the limitations, several trends in the mixed-longitudinal sample should be noted. Mean heights of the mixed-longitudinal sample of Portuguese soccer players were slightly but consistently less than the Swiss reference, while mean weights were below the reference at 11–13 years and approximated the reference at 14–18 years. Median annual increments for heights and weights of the soccer players approximated the respective medians for Swiss and United States youth. In contrast, the heights and weights of the Portuguese players were below estimated means for a composite sample of soccer players except at 18 years, but estimated increments were similar to the composite sample. Nevertheless, variation in estimated increments or rates of growth in the mixed-longitudinal sample of soccer players 11–18 years was considerable. The variation in estimated growth rates is especially relevant given the increasing emphasis on estimated increments in height over relatively short intervals, i.e., 3 to 4 months among youth soccer players at relatively young ages and the increasing dependence upon predicted estimates of maturity timing and status.

## Data Availability

The data that support the findings of this study are not publicly available due to departmental policy and privacy commitments to the study participants. Nevertheless, the data may be available upon reasonable request to Professor Manuel J. Coelho-e-Silva, Faculty of Sports Science and Physical Education, University of Coimbra, Portugal.
